# Pregnancy during adolescence: is it still a persistent concern to public health?

**DOI:** 10.1590/1806-9282.0714EDIT

**Published:** 2025-06-02

**Authors:** Denise Leite Maia Monteiro, José Maria Soares

**Affiliations:** 1 Universidade do Estado do Rio de Janeiro – Rio de Janeiro (RJ), Brazil.; 2 Centro Universitário Serra dos Órgãos – Teresópolis (RJ), Brazil.; 3 Associação Brasileirasde Obstetrícia e Ginecologia da Infância e Adolescência – São Paulo (SP), Brazil.; 4 Universidade de São Paulo, Faculdade de Medicina, Hospital das Clínicas, Departamento de Obstetrícia e Ginecologia, Disciplina de Ginecologia, Laboratório de Investigação em Ginecologia Molecular e Estrutural – São Paulo (SP), Brazil.

Pregnancy during adolescence continues to be a major challenge for health systems in Brazil and worldwide. Globally, it is estimated that each year, 16 million women between the ages of 15 and 19 years are mothers. In the poorest regions of the world, the rate of pregnant adolescents between 15 and 19 years old is four times higher than that in high-income regions. There is a higher concentration of adolescent mothers in lower economic classes and with less chance of qualifying professionally^1^. This fact is a personal plot and for society.

According to the United Nations Population Fund (UNFPA), on a global scale, the adolescent birth rate decreased from 64.5 births per 1000 women aged 15–19 years (64.5‰) in 2000 to 41.3‰ births in 2023. Although there was a reduction in all regions of the world, the largest reduction occurred in South Asia, with a slower decline in Latin America and the Caribbean, as well as sub-Saharan Africa, regions that concentrate the highest rates worldwide, with 97.9 and 51.4 births per 1000 adolescents in 2023, respectively^2^.

In Brazil, although the numbers are still worrisome, there is a significant drop in the five Brazilian regions. In 2000, one in four Brazilian babies was the child of a teenage mother. From 2001 to 2023, there was a significant reduction of 59.6% in the frequency of teenage pregnancies^3^, and currently, one in eight babies is the child of adolescents. This decline reflects the impact of public policies largely aimed at sex education, access to contraceptive methods, changes in the behavior of adolescents and their mothers/guardians, and the strengthening of primary health care.

According to the World Bank, the prevalence of adolescent pregnancy in Brazil among individuals aged 15–19 years in 2022 was 43.6%, while the highest rate in South America was in Venezuela at 82% and the lowest was in Chile at 22.8^4^. Also, Chile presents high rates of education in the population. This fact may influence the pregnancy rates in Chile.

In the week beginning February, the Week for the Prevention of Teenage Pregnancy in Brazil was celebrated, established by Law No. 13,798/2019, with the objective of disseminating information on preventive and educational measures that contribute to reducing the frequency of teenage pregnancy. One of the most important prevention factors is education. The higher the level of education, the better the chances of professional growth and new perspectives in life. Integrated sexuality education is part of the promotion of the well-being of adolescents and young people by focusing on responsible sexual behavior, respect for others, gender equality and equity, as well as the protection of untimely pregnancy, the prevention of sexually transmitted infections, and the defense against violence and abuse^5^.

The Brazilian Federation of Gynecology and Obstetrics Associations (FEBRASGO) and the Brazilian Association of Obstetrics and Gynecology of Childhood and Adolescence (SOGIA-BR) have actively participated in the National Week for the Prevention of Pregnancy in Adolescence since 2019 with educational actions in all regions of Brazil, contributing to disseminate important information to the academic community and the population.

Pregnancy during adolescence is a phenomenon related to social vulnerability, directly linked to the sociocultural, economic, and political contexts, in addition to racial and gender dimensions. Young adolescent mothers suffer a direct impact of self-esteem and mental health, which can cause frustration, anxiety, and a mixture of feelings, invisibility, fears, and concerns^6,7^. It typically occurs in poor populations, which can be influenced by poverty, lack of education, and job opportunities, and has significant impacts on the health, psychological, and socioeconomic aspects of the mother^8^. It increases the risk of low birth weight, preterm birth, mortality, preeclampsia, neonatal mortality, social isolation, delayed or neglected educational goals, and maternal depression. The social consequences include stigma, rejection, violence, and school dropout^5,7,8^.

This reality has implications not only for the health of young women but also for the social and economic development of the community, as adolescents from regions with a lower Human Development Index (HDI) still have higher rates of early pregnancy, which demonstrates the need for actions aimed at this group^9^.

Despite the sharp drop in the frequency of teenage pregnancy perceived from 2001 onward, Brazil still needs to reduce the current rate by half to approach the rates of Europe, the United States, and some countries of Asia^4^. Pregnancy between 10 and 14 years of age is generally not counted in international publications, as it represents less than 1 in every 1,000 adolescents^4^. In Brazil, pregnancy at this age only began to decrease in 2015, when 26,700 babies were born, and in 2023, this number reached 13,939 babies to mothers aged 10–14 years^3^.

However, our great concern is with the recurrence of teenage pregnancies, which remains high and stable over the years. Adolescents with a partner, little schooling, and no reproductive planning are the most likely to have two or more pregnancies before the age of 20 years, showing difficulties in postponing the first pregnancy^10^. Our research group studied the recurrence of teenage pregnancy from 2015 to 2019 and found that in the age group of 10–14 years, the drop in the period was from 5.0 to 4.7%, while among individuals aged 15–19 years, it decreased from 27.8 to 27.3%. Being married or living in a stable union increases the chance of repeated pregnancy between 10 and 14 years of age by 96% (OR 1.96) and by 40% (OR 1.40) in the age group of 15–19 years. Girls aged 10–14 years with an educational level <8 years had a 64% higher chance of repeated pregnancy (OR 1.64) and among those aged 15–19 years, the chance increased by 137% (OR 2.37)^11^.

In this scenario, the joint action of health professionals, educators, and public managers is essential. Evidence-based sex education programs, greater accessibility to contraceptive methods, and the strengthening of family and community support networks are proven effective strategies in maintaining this downward trend^6^.

Finally, for the sustainable development goals to be achieved by 2030, there must be investment in policy implementation and evaluation, as well as a focus on adolescent sexual and reproductive health. In addition, designing interventions targeting higher-risk groups, such as the less-favored family classes, through the promotion of maternal education and empowerment, is a fundamental measure to increase the reduction of teenage pregnancy^12^ and contribute to the construction of a healthier, more informed society with prospects for the future.
